# SS-A52 antigen expression in thymic carcinoma accompanied with Sjögren syndrome: A case report

**DOI:** 10.1097/MD.0000000000024491

**Published:** 2021-02-05

**Authors:** Tomomi Isono, Tomoko Wakasa, Hidenori Kusumoto, Keiji Shimada, Takafumi Ogawa, Hiroyuki Shiono

**Affiliations:** a General Thoracic Surgery; b Department of Diagnostic Pathology, Kindai University Nara Hospital, Ikoma; c Department of Diagnostic Pathology, Nara City Hospital, Nara; d Kyodo Byori Incorporation, Kobe, Hyogo, Japan.

**Keywords:** Sjögren syndrome, thoracic surgery, thymic tumor

## Abstract

**Rationale:**

The relationship between thymic tumors and Sjögren syndrome (SjS) is unknown, and surgical resection has not been optimized. Especially, thymic carcinoma with autoimmune disease is rare. Analysis of SS-A52, germinal centers, plasma cells, and Foxp3+ Treg in thymic carcinoma has never been reported, and their pathological roles in causing SjS have not been studied.

**Patient concerns:**

A 78-year-old man presented with sputum production and xerostomia while asleep. Chest computed tomography showed a homogeneous and hypodense mass in the anterosuperior mediastinum. Serum levels of the antinuclear antibody, antibody to SS-A, and antibody to SS-B were positive.

**Diagnoses:**

Thymic carcinoma (squamous cell carcinoma) and SjS.

**Interventions:**

Video-assisted thoracoscopic resection of the mediastinal tumor and postoperative radiation therapy was performed.

**Outcomes:**

The histological diagnosis was thymic squamous cell carcinoma. Histologically, the squamous carcinomatous cells were arranged in nests and cords in the fibrohyaline stroma with capsular invasion. In the stroma, dense lymphoid tissues containing large reactive germinal centers and many plasma cells were also noted. In the involuted thymus, CD20-positive mature lymphocytes infiltrated, and germinal centers were noted. Double immunohistochemical staining revealed that SS-A52 antigen was positive in both the carcinoma component and CD20-positive mature B cells. Postoperatively, the xerostomia persisted, and serum SS-A and SS-B remained positive. No evidence of carcinoma recurrence with chest computed tomography scan was observed at 18-months follow-up.

**Lessons:**

In the surgical treatment of thymic tumors with SjS, extended thymectomy might be worth considering to stop the progressive destruction of the targets of SjS-specific autoantibodies. However, the postoperative symptoms may not dramatically improve because the target organs might have changed irreversibly, and memory B cells might persist. This is the first report that demonstrated the SS-A52 antigen presentation in a thymic tumor to the best of our knowledge.

## 1 Introduction

Thymic tumors are sometimes accompanied by an autoimmune disease (AD). Myasthenia gravis (MG) remains the best-studied AD in terms of a coexisting thymic tumor. For example, approximately 10% to 15% of the patients with MG have a thymoma.^[1]^ Type-A thymoma is rarely associated with MG (0%–33%); on the contrary, MG is more commonly associated with type B1–B3 (7%–71%) thymoma.^[2]^ Morphologically, 58.8% of thymoma patients with MG (TMG) had thymic germinal centers (GC), whereas only 15.6% of the patients with thymoma but without MG showed GCs.^[3]^ GCs are specialized microstructures found within secondary lymphoid tissues that produce long-lived antibody-secreting plasma cells and memory B cells during antigen presentation by follicular dendritic cells to T cells.^[4,5]^ The number of GCs has been correlated with higher titers of antiacetylcholine receptor antibodies in MG patients. The patients showing chronicity of the GCs had persistence of the antigens, and the use of prednisolone therapy resulted in reduced GCs.^[3,6]^ In addition to thymoma, thymic follicular hyperplasia with GCs is the most common morphology in early-onset MG and is also observed in remnant thymuses adjacent to thymomas in TMG (30%–50%). Thymic follicular hyperplasia is thought to be a source of autoantibodies.^[2,6]^ This is a plausible explanation for the effectiveness of extended thymectomy (ET) as a treatment for MG. In ET, en bloc resection of the anterior mediastinal fat tissue, including the thymus, is performed. The resection borders were the diaphragm caudally, the thyroid grand orally, and the phrenic nerves laterally.^[7]^ ET for MG showed significant improvements in postoperative clinical symptoms in both nonthymomatious^[7,8]^ and thymomatous patients.^[7]^ In addition, another immunoregulatory defect is observed in MG patients: thymic and peripheral regulatory T cells (Treg) and effector T cells are functionally impaired.^[9,10]^

Despite the accumulating knowledge, the relationship between thymic carcinoma and AD remains unclear. Studies that investigated the association of thymic carcinoma with AD are rare, and only a few cases have been reported, including MG,^[11,12]^ autoimmune hepatitis,^[13]^ stiff-man syndromes,^[14]^ systemic lupus erythematosus (SLE) and hypertrophic pulmonary osteoarthropathy,^[15]^ scleroderma,^[16]^ and dermatomyositis.^[17]^ According to another recent study, 1 out of 9 patients with micronodular thymic carcinoma with lymphoid B-cell hyperplasia, had MG, whereas 1 out of 9 had Sjögren syndrome (SjS).^[18]^ In a previous report on thymic carcinoma with AD, GCs were reported.^[19]^ Surgical resection of thymic carcinoma contributed to good control in some cases of AD^[20,21]^; however, the relapse of AD was followed by postoperative metastasis of the tumor.^[22]^

Furthermore, the relationship between thymic tumors and SjS remains unknown, and the surgical resection has not been optimized. The SS-A autoantigen is an intracellular RNA-protein complex that is the target of autoantibodies present in the sera of patients with SjS and SLE.^[23,24]^ The SS-A antigenic system comprises 2 proteins: 52-kD polypeptide (SS-A52) and 60-kD polypeptide (SS-A60).^[24]^ The mRNA expression of SS-A52 and SS-A60 was higher in SjS patients than that in controls.^[25]^ SS-A52 is expressed in the cytoplasm of ductal cells of patients with SjS.^[25]^ SS-A60 is found in the cytoplasm and nucleus of acinar cells and ductal cells of patients with SjS.^[25]^ The role of these 2 polypeptides in SjS is unclear; however, antibodies, rather than autoantigens, seemed to injure the target organs. In 1 experiment, the IgG deposition and functional failure of the salivary glands following the surge of serum anti–SS-A52 antibodies after intravenous injection of SS-A52 into mice were reported. In the same report, the passive transfer of SS-A52 immune sera-induced salivary gland dysfunction.^[26]^ In another study, anti–SS-A and anti–SS-B antibodies were detected in the lacrimal fluid, and their presence in the serum or tear fluid was associated with the severity of keratoconjunctivitis sicca.^[27]^ However, the relationship between SS-A antigen and thymic tumors is still unclear. Additionally, the frequency of Foxp3+ Treg cells in salivary gland lesions in patients with SjS correlates with inflammation grade.^[28]^

We herein report a case of thymic carcinoma with GCs complicated by SjS and examine the expression and distribution of SS-A52 antigen and Foxp3+ Treg cells in the resected specimen.

## 2 Case presentation

A 78-year-old man presented to our hospital with sputum production and xerostomia while asleep. Chest computed tomography showed a homogeneous and hypodense mass, 48 mm in size with smooth contours and without contrast enhancement in the anterosuperior mediastinum. Serum levels of anti-acetylcholine receptor antibody, soluble interleukin 2 receptor, carcinoembryonic antigen, α-fetoprotein, squamous cell carcinoma–related antigen, cytokeratin 19 fragment, and human chorionic gonadotropin β-subunit were within their normal ranges. The diagnosis of thymoma was suspected. However, serologic examination revealed the following: the antinuclear antibody, antibody to SS-A, and antibody to SS-B showed positive (1:640 with a speckled pattern, 1:>240, and 1:>320, respectively); rheumatoid factor, 113 IU/ml; anti-galactose-deficient IgG antibody level, 149.5 AU/ml; serum cryoglobulin, negative. He was diagnosed with SjS, fulfilling 2 of the 4 criteria items of the revised Japanese criteria for SjS.^[29]^ Video-assisted thoracoscopic resection of the mediastinal tumor was performed. The thymic and adipose tissue around the tumor were also resected with the tumor to achieve complete resection. There was a lobulated and elastic mass in the thymus, measuring 45 × 30 mm in size. We found no pleural dissemination of the tumor to the thoracic cavity.

**Figure 1:**
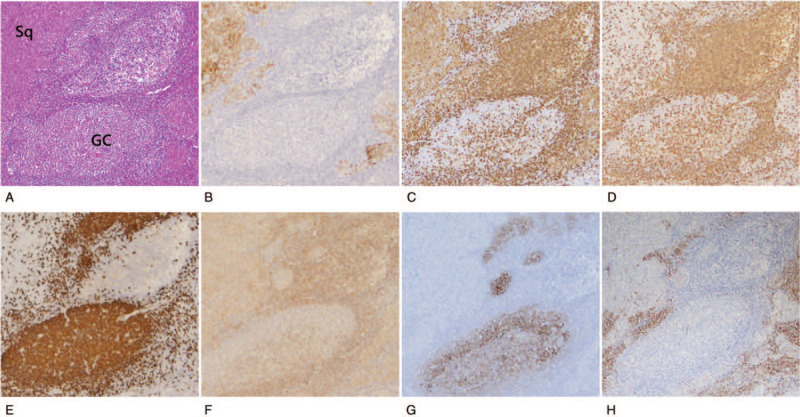
Hematoxylin-eosin staining and immunohistochemical staining of thymic Sq and GCs. (A) In hematoxylin-eosin staining, the neoplasms were composed of atypical polygonal squamous cells arranged in a sheet-like pattern and prominent GCs with defined light zone and dark zone (under 40× magnification). (B) Immunohistochemical staining for AE1AE3 is positive in epithelial cells (under 40x magnification). (C) The epithelial cells were immunoreactive to CD5, indicating thymic organ (under 40x magnification). (D) CD3 highlights T cells. (E) CD20 highlights B cells. In the stroma, T cells and B cells are arranged in a reactive pattern. CD20-positive B cells are aggregated in the GCs (under 40x magnification). (F) SS-A52 is weakly positive in the epithelial cells and infiltrated lymphocytes (under 40x magnification). (G) CD21-positive dendritic cells are arranged in the center of the GCs (under 40x magnification). (H) CD138-positive plasma cells are noted in the lymphoid stroma (under 40x magnification). GC = germinal center, Sq = squamous cell carcinoma.

This tumor was diagnosed as squamous cell carcinoma of the thymus (Masaoka stage II) based on the World Health Organization classification.^[30]^ Histologically, the neoplasms were composed of atypical polygonal epithelial cells arranged in a sheet-like pattern, and the tumor nodules were separated by abundant reactive lymphoid stroma (Fig. 1A). The epithelial cells were round to oval in shape, with large nuclei containing dense chromatin and prominent nucleoli. The mitotic count was 5 to 8/10 HPF, and keratinization was observed. The surrounding lymphoid stroma was composed of densely packed proliferating small lymphocytes, many plasma cells, and large lymphoid follicles with GCs

Immunostainings for CD3, CD5, CD20, CD21, CD138, wide-spectrum antipancytokeratin (AE1AE3), SS-A52, and Foxp3 were performed in controls (thymic squamous cell carcinoma without the autoimmune disease [Sq/AD^-^]) and, in our case (Sq+SjS), according to the manufacturer's protocols. Tumor specimens of Sq/AD^-^ were obtained from 3 patients with thymic squamous cell carcinoma without any autoimmune disease who underwent surgery around the same time in our hospital. The primary antibodies used were antihuman 52-kD Ro/SSA mouse monoclonal antibody (clone D-12; dilution rate, 1:100; Santa Cruz Biotechnology, Dallas, TX) and antihuman Foxp3 mouse monoclonal antibody (clone 236A/E7; dilution rate, 1:100; eBioscience Inc., San Diego, CA). To confirm the expression of SS-A52, a dual-fluorescent staining for SS-A52/CD3, SS-A52/CD20, and SS-A52/AE1AE3 was also performed manually in our case using the Opal 4-color manual IHC kit (Akoya Biosciences, San Francisco, CA), according to the manufacturer's protocols. For nuclear staining, 4’,6-diamidino-2-phenylindole (DAPI) was also stained and shown in blue. The reaction product was fluorescently labeled with Opal 520 and Opal 690 fluorophore working solution (Opal 4-color manual IHC kit; Akoya Biosciences) and was observed under a fluorescent microscope BZ-X810 (Keyence Corporation, Osaka, Japan).

**Figure 2:**
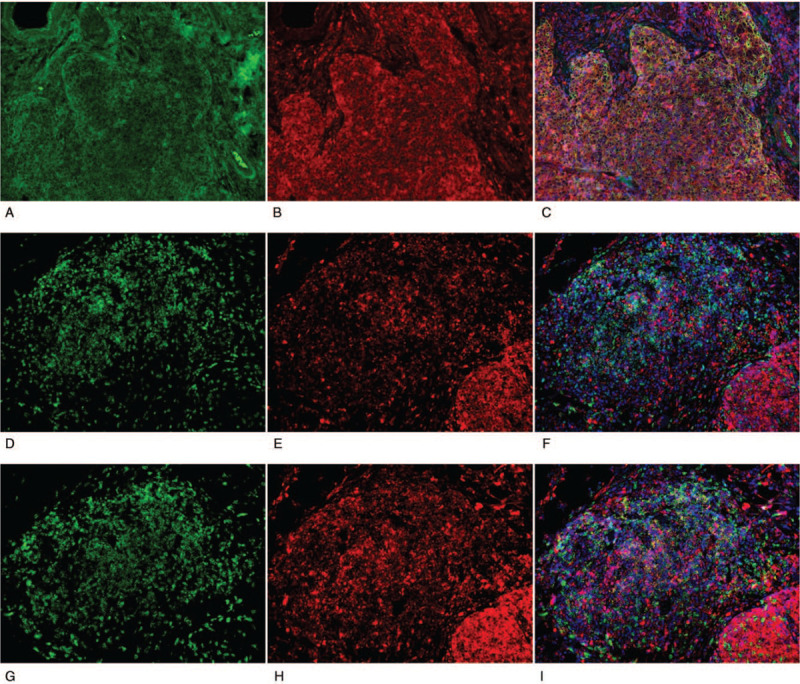
Immunohistochemical double staining of the tumor of thymic squamous cell carcinoma accompanied with Sjögren syndrome. (A-C) AE1AE3 and SS-A52 show partial co-expression in the GCs and tumor. (A) Green, AE1AE3 (under 200× magnification);(B) red, SS-A52 (under 200× magnification); (C) yellow, co-expression of AE1AE3 and SS-A52 in a merged image with DAPI (blue). (under 200× magnification). (D-F) CD3 and SS-A52 do not show co-expression in the GCs and tumor. (D) Green, CD3 (under 200× magnification); (E) red, SS-A52 (under 200× magnification); (F) yellow, co-expression of CD3 and SS-A in a merged image with DAPI (blue). No coexpression of SS-A52 and CD3 can be observed. (under 200× magnification). (G-H) CD20 and SS-A52 show partial co-expression in the GCs and tumor. (G) Green, CD20 (under 200× magnification); (I) red, SS-A52 (under 200× magnification); (H) yellow, co-expression of CD20 and SS-A52 in a merged image with DAPI (blue). Coexpression of CD20 and SS-A52 can be partially observed. (under 200× magnification). DAPI = 4′,6-diamidino-2-phenylindole, GC = germinal center.

**Figure 3:**
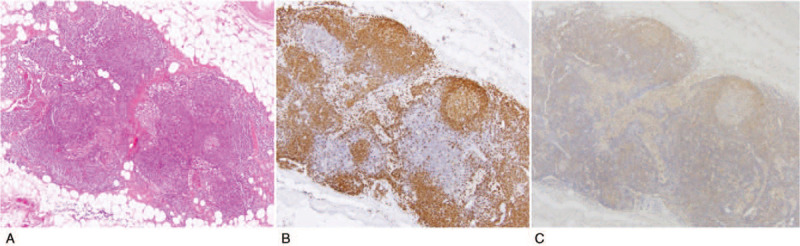
Involuted thymus. (A) Hematoxylin-eosin staining of the involuted thymus shows age-related architectural alteration border directly on the adipocytes. GCs are noted (under 40× magnification). (B) Immunohistochemical staining for CD20 is strongly positive in the GCs and the lymphocytes aggregated in the surrounding involuted thymus (under 40× magnification). (C) SS-A52 is weakly positive in the GCs and the lymphocytes aggregated in the surrounding involuted thymus (under 40× magnification).

**Figure 4:**
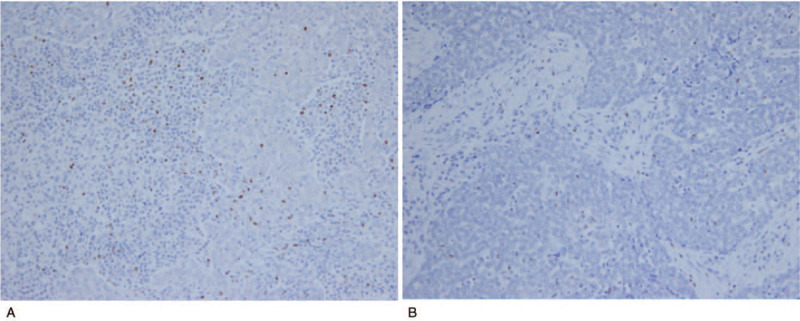
Immunohistochemical staining for Foxp3. (A) In the case of thymic squamous cell carcinoma with Sjögren syndrome, Foxp3-positive lymphocytes are detected in the carcinoma component and lymphoid stroma (under 200× magnification). (B) Thymic squamous cell carcinoma without autoimmune disease. A few lymphocytes infiltrate the carcinoma component and fibrous stroma but are negative for Foxp3 (under 200× magnification).

Immunohistochemical studies showed that the carcinoma component was positive for AE1AE3 and CD5 (Fig. 1A-C). The surrounding lymphoid stroma contained CD3-positive small lymphocytes (Fig. 1D). CD20-positive B lymphocytes were seen in the center of GCs and surrounding lymphoid stroma in reactive pattern (Fig. 1E). SS-A52 was diffusely positive in the cytoplasm of the carcinoma cells (Fig. 1F). In the center of the GCs, there were small foci of CD21-positive dendritic cells (Fig. 1G). CD3-positive T lymphocytes and numerous CD138-positive plasma cells were observed in the interfollicular area (Fig. 1H). In the dual-fluorescent staining for SS-A52/AE1AE3, SS-A52 was positive in tumor cells that were also positive for AE1AE3 (Fig. 2A-C). In the dual-fluorescent staining for SS-A52/CD3 and SS-A52/CD20, the co-expression of SS-A52 and CD20 was partially observed, but that was not the case with SS-A52 and CD3 (Fig. 2D-I). In the involuted thymus surrounding the carcinoma, prominent GCs were noted, and the aggregated lymphocytes were mainly CD20-positive B cells that were weakly positive for SS-A52 (Fig. 3). In contrast, GC formation and SS-A52 positivity were not observed in Sq/AD^-^ (data not shown). Foxp3-positive lymphocytes infiltrated the squamous cell carcinoma and lymphoid stroma (Fig. 4A). In Sq/AD^-^, few lymphocytes infiltrated the carcinoma component and fibrous stroma, but they were negative for Foxp3 (Fig. 4B).

The postoperative course was uneventful, and the patient underwent adjuvant radiation therapy with a total dose of 50 Gy divided into 30 fractions. After radiation therapy, cevimeline hydrochloride hydrate (30 mg/day) was started, and xerostomia improved slightly. At the 18-month follow-up, no evidence of tumor recurrence was detected. However, serum levels of the antinuclear antibody, SS-A antibody, SS-B antibody, and rheumatoid factor remained unchanged. Both the Saxon test and Schirmer test remained positive.

## 3 Discussion

In this report, a specific autoantigen was found in the tumor tissues of a patient with thymic squamous cell carcinoma with SjS. We believe that the autoantigen-expressing tumor tissues may be the culprit for SjS. Moreover, the non-resected involuted thymus may still be responsible for the postoperative SjS because B-cell infiltration was found. Therefore, ET may be useful for thymic tumors with SjS to normalize the postoperative serum anti–SS-A antibody levels. This is the first report to reveal positive SS-A52 expression in thymic carcinoma via immunohistochemistry staining to the best of our knowledge.

In our patient, the distribution patterns of CD3 and CD20 were relatively compartmented, and CD21 showed a network pattern of GC. They are considered similar structures of active GCs in the lymph nodes.^[31]^ However, Foxp3-positive lymphocytes were found in the tumor cells and among infiltrated lymphocytes of Sq+SjS. Although the Treg cells may be activated to repress the abnormal immune responses, they might not prevent SjS. Based on these findings, SS-A52 might be expressed by the tumors, and the autoantibodies might be produced by the B cells in extranodal GCs, which might be enough to destroy the local exocrine glands or skin and cause the SjS.

Interestingly, our case showed similar findings to those of TMG on immunohistochemistry staining: peripheral lymphocytes infiltration and GCs. Abnormal T-cell functionality, probably caused by the inflammation of the tumor and B-cell infiltration to organize GCs, has been observed in the thymus with early-onset MG.^[32]^ Recent research showed that the titers of anti-acetylcholine receptor antibody were significantly higher in TMG patients with numerous thymic GCs than in TMG patients with fewer GCs, supporting the hypothesis that thymic GCs were associated with MG in thymoma patients.^[3]^ A similar pathogenesis might exist in the thymic tumors with SjS. We reviewed past reports on thymic tumors accompanied with SjS in patients who underwent surgery (n = 16, Table 1). Lymphoid follicles were observed in 7 out of the 16 cases (no descriptions and no images of GCs in 5 cases), and other complications, including MG, pure red cells aplasia, and so on, were reported in 3 out of 7 cases. The extranodal GCs in these 7 cases might also contribute to other ADs.

**Table 1:** Case reports on surgeries performed on thymic tumors with SjS.

										Postoperative course	
Year and reference	Country	Sex	Age (years)	Diagnosis	Lymphoid follicle in the tumor or thymus	Operation	Chemotherapy, prednisolone, or other medication	Radiation	Complication	SjS	Tumor
1964^[33]^	UK	F	56	Thymoma	Not found in image	Thymectomy	Unknown	Unknown	Henoch-Schönlein purpura	No change	Unknown
1975^[34]^	Japan	M	50	Lymphoepithelioma	No explanation and no image	Thymectomy	Prednisolone	No	Myasthenia	Improvement in the swelling of the parotid gland after prednisolone administration	Unknown
1980^[35]^	Japan	F	27	Thymoma	Not found in image	Thymectomy	Prednisolone	No	Hyperviscosity syndrome (hypergammaglobulinemia)	No change	Unknown
1984^[36]^	Japan	F	36	Thymoma	Yes	Thymectomy	Plasma exchange and Prednisolone	No	Pulmonary embolism, hypergammaglobulinemia	Unknown	Unknown
1987^[37]^	USA	M	43	Malignant thymoma	No explanation and no image	Partial resection of tumor	No	Yes	No description	Worsened	Unknown
1991^[38]^	Japan	F	58	Malignant thymoma	Not found in image	Thymectomy	Prednisolone	Yes	Diffused pan bronchiolitis	Unknown (died of pneumonia)	Unknown (died of pneumonia)
1993^[39]^	France	F	40	Lymphoepithelial thymoma	Yes	Thymectomy	Cyclophosphamide and prednisolone	50 Gy	Bronchiolitis	Worsened	Unknown
1994^[20]^	Japan	F	73	Thymic carcinoma (Sq)	Not found in the thymus	ET, resection of the pericardium	Prednisolone	50 Gy	Hashimoto disease, dermatomyositis	No change	No recurrence
1996^[40]^	Japan	F	19	Lymphoepithelial thymoma	Yes	Complete resection of thymoma	Prednisolone	No	No description	Xerostomia reappeared after 1 month of surgery	No recurrence
1996^[41]^	Japan	F	43	Thymoma	Yes (in the tumor)	Thymectomy	Cyclophosphamide, cisplatin doxorubicin hydrochloride, and vincristine sulfate (preoperative) Prednisolone	No	Anemia	No change	Unknown
1998^[12]^	Israel	F	72	Thymic carcinoma	No explanation and no image	Thymectomy	Cisplatinum	No	No description	No change	No recurrence
1999^[42]^	USA	F	63	Type AB thymoma	No explanation and no image	Thymectomy	Intravenous gammaglobulin	No	Sensory neuropathy, hypogammaglobulinemia	Unknown	Unknown
2001^[43]^	Japan	F	36	Type B1 thymoma	Yes (in the thymus)	Surgical resection of thymoma, the thymus, and part of the pericardium	Prednisolone, cyclosporin	40 Gy	Myasthenia gravis, pure red cell aplasia	No change	No recurrence
2010^[44]^	Taiwan	F	66	Thymic carcinoma (Sq)	Yes (in the tumor)	Radical thymothymectomy	No	54 Gy	Syndrome of inappropriate of antidiuretic hormone	Slightly improved	No recurrence
2013^[45]^	Taiwan	M	63	Type A thymoma	No explanation and no image	Thymectomy	No	No	Myasthenia gravis	Improved	No recurrence
Ours	Japan	M	78	Thymic carcinoma (Sq)	Yes (in the tumor and thymus)	Thymectomy	No	50 Gy	None	No change	No recurrence

ET = extended thymectomy, F = female, M = male, SjS = Sjögren syndrome, Sq = squamous cell carcinoma.

ET for thymic tumors with SjS may normalize the postoperative serum autoantibodies and stop the progression of SjS, but it does not always improve the symptoms. First, ET may reduce the incidence of postoperative autoantibodies. In our case, the postoperative serum level of anti–SS-A antibody was positive. The non-resected thymus may be the culprit because GCs, B-cell infiltration, and SS-A52 expression were found in the surrounding involuted thymus. If the hypothesis that autoantibody injures the target organs is true, then ET may reduce the number of B cells, normalize serum autoantibodies, and stop further progression of SjS.

Second, another plausible explanation for the persisting symptoms could be the irreversible destruction of the exocrine glands. In a recent study (n = 66 vs n = 67), B-cell depletion by rituximab in patients with SjS showed no clinical improvement in dry mouth, salivary, and lacrimal flow rates, among others.^[46]^ This result may be explained by the fact that the function of the exocrine glands was irreversibly destroyed in many cases. In previous case reports on thymic tumors, postoperative improvement of the symptoms of SjS was found in only 2 out of the 7 cases with no recurrence of tumor, as shown in Table 1. We speculate that the surgery might have been performed at a relatively early stage of SjS in those 2 cases and at a progressive stage close to irreversible in the remaining 5 cases without improvement. Therefore, ET of thymic tumors with SjS may normalize the serum autoantibodies and stop the progression of SjS yet not improve the symptoms. To examine this hypothesis, ET and following the postoperative clinical course and autoantibodies are necessary. However, ET was not performed on this patient because we had no evidence of any benefit of resecting the thymus when we performed the surgery, and the patient refused further surgery. Hence, more case studies are necessary. Another plausible reason for persisting postoperative SjS might be existing memory B cells.

## Acknowledgments

We would like to thank Editage (www.editage.com) for English language editing.

## Author contributions

**Conceptualization:** Tomomi Isono, Tomoko Wakasa, Hidenori Kusumoto, Keiji Shimada.

**Data curation:** Tomomi Isono, Tomoko Wakasa, Keiji Shimada, Takafumi Ogawa, Hiroyuki Shiono.

**Formal analysis:** Tomomi Isono, Tomoko Wakasa.

**Funding acquisition:** Hiroyuki Shiono.

**Investigation:** Tomomi Isono, Tomoko Wakasa, Takafumi Ogawa.

**Methodology:** Tomoko Wakasa, Hidenori Kusumoto, Takafumi Ogawa, Hiroyuki Shiono.

**Project administration:** Hiroyuki Shiono.

**Resources:** Keiji Shimada.

**Software:** Takafumi Ogawa.

**Supervision:** Hiroyuki Shiono.

**Validation:** Tomomi Isono.

**Visualization:** Tomomi Isono, Takafumi Ogawa.

**Writing – original draft:** Tomomi Isono, Tomoko Wakasa, Takafumi Ogawa.

**Writing – review & editing:** Tomomi Isono, Tomoko Wakasa, Hiroyuki Shiono.

## Corrections

The corresponding author email was originally misspelled as hshino@med.kindai.ac.jp and has been corrected to hshiono@med.kindai.ac.jp.

In the 1984 row of Table 1, the Chemotherapy, prednisolone or other medication column appeared incorrectly as plasma exchange and has been corrected to plasma exchange and prednisolone.
